# Characteristics of ankle kinematics in patients with ankle instability with or without an osteochondral lesion of the talus

**DOI:** 10.3389/fspor.2025.1708093

**Published:** 2025-12-08

**Authors:** Longzhou Hua, Chenglin Wu, Ling Zhang, Longxiang Li, Zhongmin Shi, Shaobai Wang

**Affiliations:** 1 Key Laboratory of Exercise and Health Science of the Ministry of Education, Shanghai University of Sport, Shanghai, China; 2 Department of Orthopedics, Shanghai Sixth People’s Hospital Affiliated to Shanghai Jiao Tong University, Shanghai, China

**Keywords:** ankle instability, osteochondral lesion of the talus, gait kinematics, 6-DOF kinematics, treadmill walking, range of motion

## Abstract

**Purpose:**

This study aimed to investigate ankle kinematic alterations in patients with chronic ankle instability (CAI), with or without a concomitant osteochondral lesion of the talus (OLT), during level walking. Unlike previous studies that did not distinguish between isolated CAI and CAI with an OLT, this study used MRI-confirmed OLT diagnosis combined with six-degrees-of-freedom motion analysis to identify OLT-specific kinematic patterns, which may provide novel biomechanical markers to facilitate clinical screening and early diagnosis.

**Methods:**

A total of 33 patients with CAI (15 with an OLT and 18 without an OLT) and 18 healthy controls were enrolled. Ankle kinematics were assessed using a joint motion analysis system during treadmill gait at a self-selected speed. Statistical parametric mapping (SPM) was used to compare kinematic patterns across groups.

**Results:**

Compared to healthy subjects, the patients with OLTs demonstrated reduced plantarflexion during the early stance (1%–5%, 9%–10%), initial (67%–83%), and terminal swing (91%–100%) phases of the gait cycle, and the patients with isolated CAI demonstrated reduced distal translation during the initial swing (65%–69%) phase of the gait cycle. The patients with OLTs exhibited decreased a range of motion (ROM) in plantarflexion (9.6°, *P* < 0.001), internal rotation (3.0°, *P* = 0.01), and lateral translation (0.3 mm, *P* = 0.02); in contrast, the patients with isolated CAI showed reduced inversion rotation (3.1°, *P* = 0.009) and lateral translation (0.4 mm, *P* < 0.001) compared with healthy controls.

**Conclusion:**

The presence of osteochondral lesions further exacerbates kinematic abnormalities in ankles with CAI during gait. These distinct kinematic signatures—particularly reduced plantarflexion ROM in patients with OLTs vs. reduced inversion ROM in patients with isolated CAI—may serve as objective clinical markers to distinguish between these conditions, potentially reducing misdiagnosis rates and enabling clinicians to implement targeted rehabilitation strategies to prevent progression to ankle osteoarthritis.

## Introduction

Chronic ankle instability (CAI) has been observed to follow the occurrence of an initial acute inversion sprain of the ankle (1). Most people with a history of ankle sprains will suffer at least one additional sprain (2, 3) and develop residual symptoms collectively known as CAI, which includes a feeling of instability, pain, giving way, and ankle swelling. It has been estimated that 38%–41% of ankle sprains result in CAI, whilst half of all ankle sprains reportedly result in an osteochondral lesion of the talus (OLT), and a concomitant OLT is estimated in one-third of patients with CAI. These data are likely an underestimation, as a significant proportion of cases receive neither supervised nor professionally administered care (4–6). However, the absence of specific symptoms for an OLT frequently results in misdiagnosis as a simple ankle sprain or CAI in clinical settings, affecting the clinical regimen (7, 8). Additionally, CAI is well documented to disrupt the ankle's usual alignment during kinematic and static movements (9). This disruption allows the talus to move forward and internally rotate excessively relative to the tibia (10). In addition, extant research suggested that the majority (59%–78%) of OLTs are located on the medial side of the talus (the posterior medial zone and central medial side) (11, 12), indicating that a biomechanically induced mechanism of OLT may be associated with CAI.

Previous studies have investigated biomechanical alterations in patients with CAI, including in force transmission patterns (13), balance of intrinsic muscle strength in the ankle joint, delayed muscle activation (particularly in the tibialis anterior and peroneal muscles) (14–16), and the kinematics manifest during disparate movement patterns, which collectively compromise dynamic ankle stability (17, 18). Recent advances in musculoskeletal modeling have further revealed that patients with CAI exhibit altered muscle synergy patterns and compensatory strategies during dynamic tasks in order to maintain functional performance, reflecting neuromotor reorganization following injury (19, 20). Nevertheless, studies have not distinguished between those with isolated CAI and those with OLTs when including subjects. It can thus be hypothesized that this uncertain variable contributes to the discrepancy in biomechanical findings in patients with CAI (17, 18). Gait analysis has become an important clinical tool for assessing pathologies manifested by gait abnormalities (21). A few studies have indicated that an OLT manifests gait characteristics that are distinct from those of CAI alone, attributable to articular malalignment. Cao et al. (22) provided preliminary evidence by investigating the biomechanical changes in stair descending in patients with CAI (with an OLT or not) and found that maximal plantarflexion in patients with OLTs was significantly smaller than that in patients with CAI and in healthy subjects; furthermore, maximal internal rotation was smaller than that in patients with CAI. Nevertheless, the ankle gait characteristics of CAI alone or in combination with an OLT have not been confirmed during level walking, the most common of daily activities.

Methodological limitations, namely, the lack of imaging confirmation, may explain the inconsistent kinematic findings reported across studies. Therefore, this current study aimed to measure the gait kinematic characteristics of the ankle joint in patients with CAI with or without an OLT and to identify biomechanical targets for clinical diagnosis and rehabilitation interventions. We measured ankle six-degrees-of-freedom (6-DOF) kinematics in patients with CAI in treadmill gait with and without an OLT using a joint motion function analysis system. We hypothesized that the CAI and OLT group would exhibit reduced ankle plantarflexion during the early stance and swing phases.

## Methods

This study was conducted according to a protocol approved by the Institutional Review Board. An *a priori* power analysis was completed using G*power (version 3.1.9.7) to determine the sample size needed to detect significant differences among the groups. The maximum flexion angle was selected as the primary kinematic parameter. The average flexion angle was found to be 7.88° for the patients with CAI and an OLT, 12.33° for the patients with isolated CAI, and 10.07° for the healthy group. The power (1-β) was set to 0.9. The significance level was set at 0.05. The results showed that a sample size of 11 ankles in each group could detect kinematic differences between the three groups, with a significance level of 0.05. Therefore, the number of subjects recruited in this study (15 ankles in the CAI and OLT group, 18 ankles in the isolated CAI group, and 18 ankles in the healthy group) met the sample size requirement.

A total of 33 subjects with CAI (15 with an OLT and 18 without an OLT) and 18 healthy subjects were included in this study based on inclusion and ranking criteria adapted from the recommendations of the International Ankle Consortium (23). Overall, 15 patients with CAI and an OLT [six women and nine men, mean age 37.3 years (SD 7.6), mean height 171.1 cm (SD 7.6), mean weight 76.1 kg (SD 18.0)] were included. The criteria for an OLT were based on magnetic resonance imaging (MRI) with T1ρ mapping sequences. An OLT was diagnosed when T1ρ relaxation time exceeded 45 ms in the talar cartilage or subchondral bone, indicating cartilage matrix degeneration (24). Furthermore, the patients underwent MRI within 1 year (mean 5 months) after the ankle injury. Moreover, 18 CAI patients without an OLT were included in this study [seven women and 11 men, mean age 35.2 years (SD 8.3), mean height 168.8 cm (SD 10.3), mean weight 73.4 kg (SD 16.2)]. Inclusion criteria for CAI (without OLT) were as follows: (1) first unilateral ankle sprain within 1 year, (2) MRI showing injury to the lateral ligament of the ankle, and (3) feeling of “giving way” and sensation of ankle instability. Finally, 18 healthy subjects [seven women and 11 men, mean age 35.3 years (SD 4.1), mean height 168.4 cm (SD 8.2), mean weight 68.9 kg (SD 9.9)] were enrolled, comprising volunteers without underlying foot deformities and musculoskeletal disorders. These volunteers were recruited locally through a public website to ensure they were age-, height-, and weight-matched with the patients.

**Figure 1:**
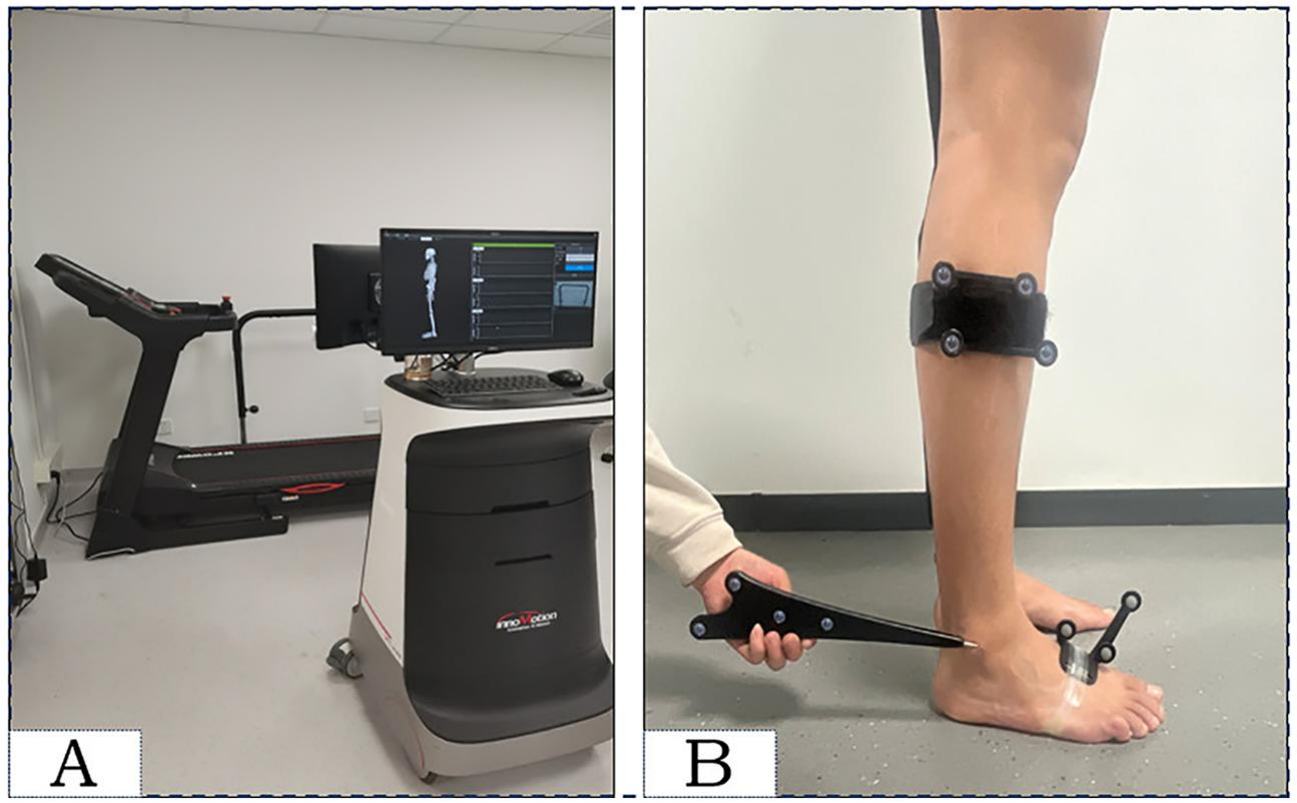
The instrumentation configuration **(A)** and computed spatial three-dimensional positions of the ankle joints, illustrated with the probe directed towards the lateral ankle **(B)**.

Motion data were collected using a motion capture system (Opti-Ankle; Innomotion Inc., Shanghai, China) at 60 Hz (Figure 1A). The system was dynamic, real-time, three-dimensional, and objective, with a footprint of only 4.0 m x 2.0 m x 2.5 m. The reliability of this system for measuring lower extremity 6-DOF kinematic parameters has been verified. In the context of walking, it is consistent; specifically, the intra-rater intra-class correlation coefficient (ICC) for rotational parameters can reach 0.9, the inter-rater ICC can reach 0.99, and the ICC values for translational parameters can reach 0.93 and 0.96, respectively (25). Two infrared optical trackers were affixed onto the subject's lower leg and dorsum of the foot with straps, and the spatial 3D trajectory of the infrared light-reflecting markers (OK-Marquer; Innomotion Inc.) was captured by a dual-head stereo infrared camera with a frequency of 60 Hz at a tracking accuracy of 0.3 mm root mean square (RMS) (26). A handheld digitizing probe was used to identify the bony landmarks to define the horizontal plane (Figure 1B) (27). The previously mentioned infrared camera was used to capture the subject’s adaptive velocity walking motion information and customized software (Optimum, Innomotion Inc.) was used to determine the gait cycle and calculate the 6-DOF kinematic parameters of the ankle joints (28).

The handheld probe was used to identify tibial and ankle anatomic landmarks (medial epicondyle of the femur, lateral epicondyle of the femur, medial malleolus, lateral malleolus, second metatarsal, and the ground-neutral position) in a neutral standing position. This static position was used to establish the initial anatomical frame of reference. Ankle joint kinematics in 6-DOF were captured at 60 Hz in 15-s walking trials during walking at an adaptive speed on a treadmill after the subjects had warmed up (5 min of treadmill gait training) to ensure the motion patterns were similar to those observed in their ground gait (29). The researchers calculated and compared ankle kinematics for each subject using approximately 20 gait cycles on a treadmill to reduce experimental errors.

**Figure 2:**
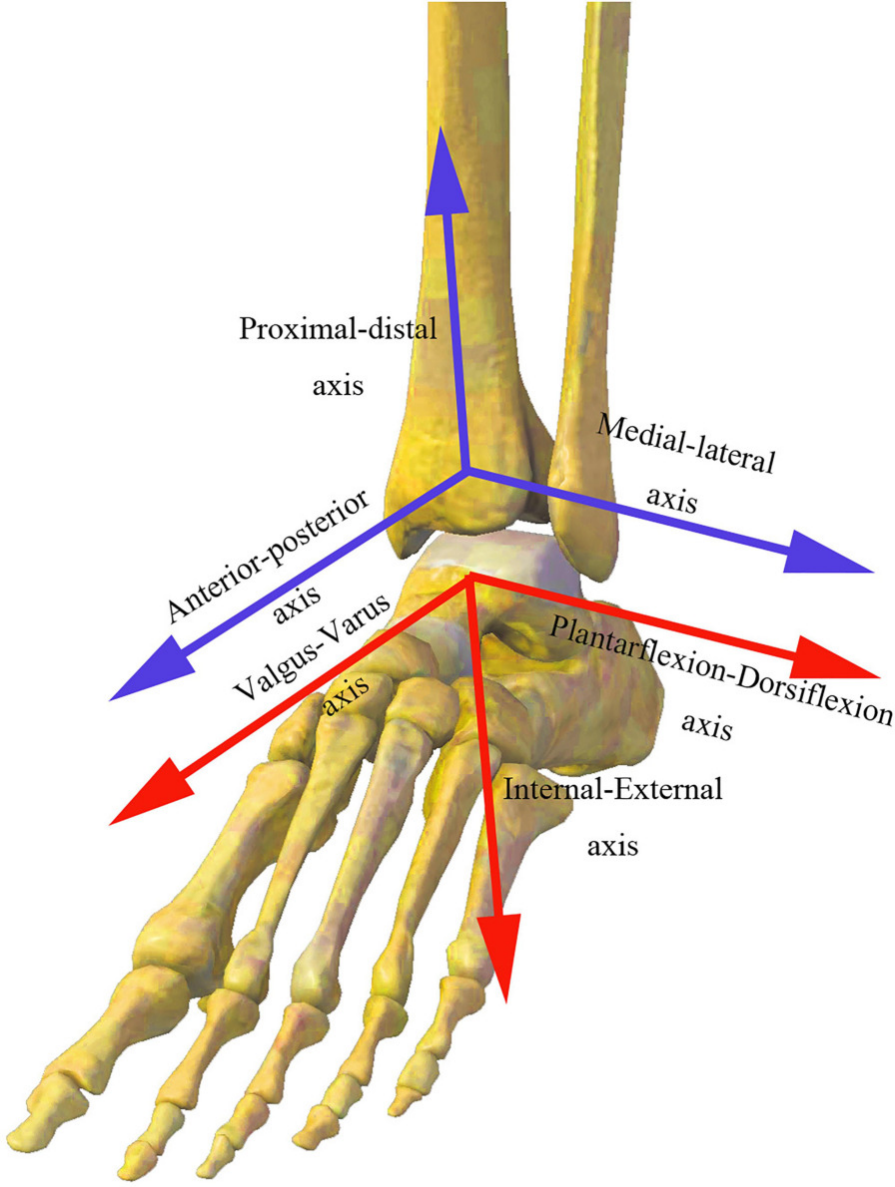
Definitions of the ankle joint coordinate systems.

The spatial 3D position of the ankle joint was calculated in each frame of gait based on the geometric relationship between the anatomical positions of the tibia and the ankle complex identified by the handheld probe and the rigid tracking plates at the calf and ankle. The kinematic parameters of the joint include flexion and extension, internal and external rotation, and inversion and eversion, as well as anteroposterior translation, medial and lateral translation, and superior and inferior translation of the ankle joint complex relative to the tibia in the sagittal, horizontal, and frontal planes (Figure 2). Each subject’s walking data were normalized to a 100-point gait cycle from a heel strike to the next heel strike, and the standardized gait cycle was then divided into a stance phase (0%–62%) and a swing phase (63%–100%) (30).

Ankle kinematic curves were compared across the standardized time series (including the stance and swing phases of the gait cycle) using ANOVA with *post-hoc* t-tests in one-dimensional statistical parametric mapping (SPM). All the SPM analyses were implemented in MATLAB 2017a (The MathWorks Inc., USA). Gaussian random field theory (inherently accounting for temporal autocorrelation and providing family-wise error control) was used to make statistical inferences and calculate thresholds for SPM[F] and *post-hoc* SPM[t]. Any cluster of SPM[t] exceeding this threshold was considered significantly different. The range of motion (ROM) in six DOF was analyzed using one-way ANOVA in SPSS (version 27.0; IBM, USA). Prior to the ANOVA, the normality of the data was assessed using the Shapiro–Wilk test, and homogeneity of variance was evaluated using Levene's test. For ROM comparisons, all the variables met parametric assumptions. The differences were regarded as statistically significant when *p* < 0.05.

## Results

### Ankle kinematics

**Figure 3:**
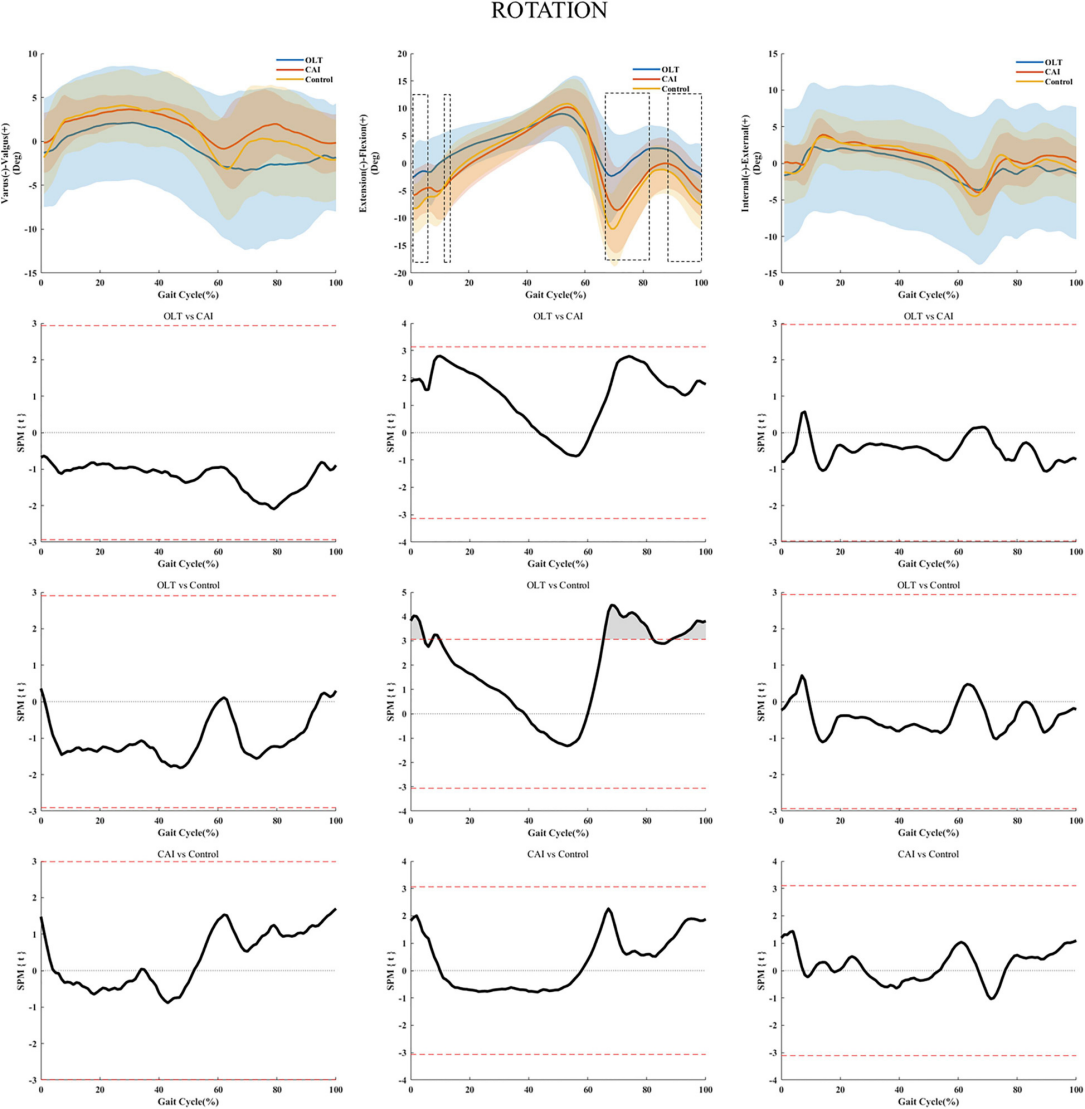
Ankle kinematics during the walking gait cycle. **(A–C)** Mean ankle valgus/varus, dorsiflexion/plantarflexion, and internal/external rotation angle trajectories and standard deviations during the walking gait cycle. Three-column SPM analyses show that only one comparison (plantarflexion/dorsiflexion) reached statistical significance, as indicated by the red dotted lines above and below 0. OLT, patients with chronic ankle instability and osteochondral lesions of the talus; CAI, patient with chronic ankle instability; Control, healthy subjects. The boxed area represents the area of statistical significance between the patients with CAI and an OLT and healthy people. Deg, degree.

**Figure 4:**
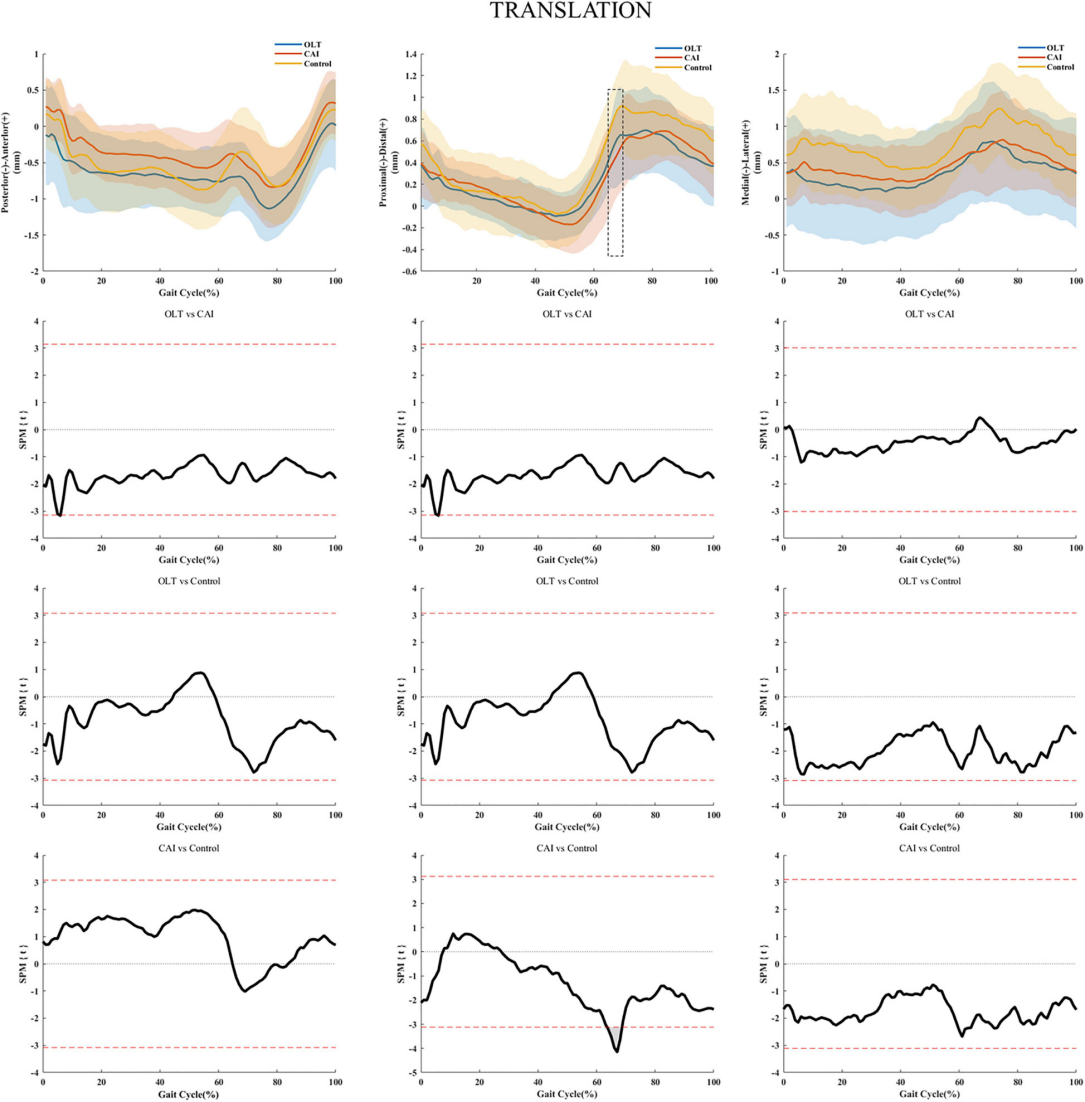
Ankle kinematics during the walking gait cycle. **(A–C)** Mean anterior-posterior, proximal-distal, and medial-lateral ankle translation trajectories and standard deviations during the walking gait cycle. Three-column SPM analyses show that only one comparison (proximal/distal) reached statistical significance, as indicated by the red dotted lines above and below 0. OLT, patients with chronic ankle instability and osteochondral lesions of the talus; CAI, patient with chronic ankle instability; Control, healthy subjects. The boxed area represents the area of statistical significance between patients with CAI and healthy people. mm, millimeter.

During the early stance (1%–5%, 9%–10%), initial (67%–83%), and terminal (91%–100%) swing phases of the gait cycle, the patients with OLTs showed less plantarflexion of the ankle joint compared with healthy subjects (Figure 3). During the initial (65%–69%) swing phase of the gait cycle, less distal translation of the ankle joint was found in the patients with CAI than in the controls in the horizontal plane (Figure 4). There were no statistically significant differences in ankle kinematics for the other directions.

### Range of motion

**Figure 5:**
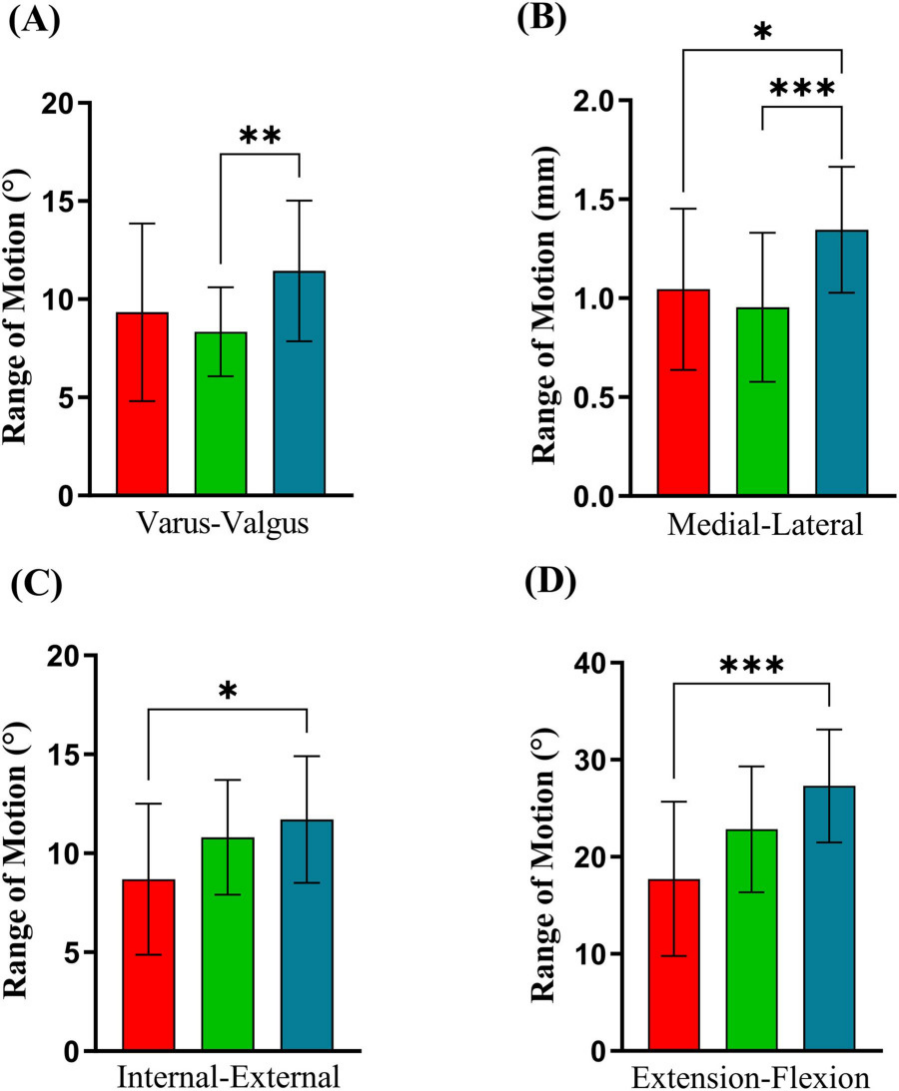
Range of motion (mean ± SD) of the kinematic parameters of the OLT (red column), CAI (green column) and control (blue column) during the treadmill gait test. **(A)** Valgus/varus, **(B)** medial/lateral, **(C)** internal/external, **(D)** plantarflexion/dorsiflexion. *Statistically significant difference (*P* < 0.05), **Statistically significant difference (*P* < 0.01), ***Statistically significant difference (*P* < 0.001). °, degree; mm, millimeter.

The valgus/varus ROM during the gait cycle of the ankles in the isolated CAI groups was significantly smaller than that of the ankles in the healthy group, with an ROM reduction of 3.1° [SD1.0, (95% CI−5.1 to −1.1, *P* = 0.003)] (Figure 5A). In addition, the ROM in internal/external rotation in the CAI and OLT group was significantly less than that of the healthy controls, with a mean ROM reduction of 3.0°[SD1.0, (95% CI−5.0 to −1.0, *P* = 0.004)], (Figure 5C). Interestingly, this study also found that the ROM in ankle plantarflexion/dorsiflexion of the CAI and OLT group was significantly less than that of the healthy group, with a mean ROM reduction of 9.6°[SD2.0, (95% CI−13.6 to −5.6, *P* < 0.001)], (Figure 5D). No significant difference was found in 3D translation, except for medial/lateral translation, with a reduced ROM of 0.3 mm (SD0.1, (95% CI−0.5 to 0.1, *P* = 0.007) in the CAI and OLT group and 0.4 mm (SD0.1, (95% CI−0.6 to 0.2, *P* < 0.001) in the isolated CAI group (Figure 5B).

## Discussion

This study investigated the 6-DOF kinematics of CAI ankles with or without an OLT during treadmill gait. In this study, reduced ankle plantarflexion during the early stance and initial swing gait phases in patients with an OLT, and reduced ankle distal translation in the initial swing phase in patients with isolated CAI were detected. Furthermore, significant statistical differences were detected in rotation and translation among the three groups.

Physical examination and imaging are critical for the diagnosis and characterization of OLTs, yet no universally accepted optimal diagnosis and treatment have been established (7). Some osteochondral lesions of the ankle may share similar pathophysiological mechanisms, such as eccentric loading on the medial talus or tibia, resulting in cartilage injury or adaptive remodeling (5). These lesions are often post-traumatic, with MRI being the preferred diagnostic modality (24). Previous kinematic studies of patients with CAI have not incorporated MRI or arthroscopic evaluations, limiting their ability to distinguish coexisting OLTs and contributing to inconsistent findings (17, 18, 31). MRI was used to confirm whether there was a concomitant OLT in patients with CAI in this study, providing novel insights into how OLTs alter ankle biomechanics beyond the effects of isolated CAI. This study showed that patients with OLTs had reduced ankle plantarflexion during the early stance (1%–5%, 9%–10%), initial swing (67%–83%), and terminal swing phases (91%–100%) of the gait cycle when walking compared with healthy subjects. Patients with OLTs have an increased reliance on bony stability to avoid ankle re-sprains (32) and adopt a pain-relieving walking strategy, because decreased plantarflexion can reduce contact pressure on the OLT and alleviate articular pain (33, 34). Additionally, an increased ankle dorsiflexion moment during the stance phase of the gait was found in 16 subjects with CAI (eight without an OLT and eight with OLTs) during stair descent using the AnyBody Modeling System by Cao et al. (35), which also explains the decreased plantarflexion in the patients with OLTs.

Our results also suggested that patients with isolated CAI had reduced ankle distal translation during the initial swing phase (65%–69%) of the gait cycle when walking compared with healthy subjects. Unlike previous studies, this study showed that individuals with CAI exhibited reduced ankle plantarflexion during the swing phase, which may be attributed to the fact that previous studies’ CAI models or inclusion criteria did not account for perceived pain. However, some studies found a high incidence of pain in patients with CAI during daily activities, which may be related to the reduced ankle plantarflexion observed in this study (36). Furthermore, an alteration of the motor control strategy of the central nervous system in patients with CAI may explain the observed differences in ankle kinematics (22). Electromyography (EMG) activity has shown that delayed anterior tibial and peroneal activation occurs in patients with CAI, and the reduced ankle plantarflexion during gait leads to limited distal movement in these patients (35). Patients with CAI exhibit reduced ankle distal translation in the initial swing phase because the captured rigid body motion trajectory was projected closer to the center of the ankle complex on the tibial prolongation line when the ankle has reduced plantarflexion. Hypoactivation of the tibialis anterior and fibularis muscles and an anterior talofibular ligament (ATFL) rupture exacerbate the excessive anterior translation of the talus, allowing the cartilage of the posterior tibial margin to wear out, and the softer talus cartilage is more susceptible to wear (37).

Consistent with earlier research (17, 22, 33), this study has observed decreased ankle ROM in patients with CAI, with or without OLT, in comparison to the healthy subjects. However, this study goes a step further by identifying OLT-specific patterns of ROM reduction. The patients with OLTs demonstrated significant reductions in plantarflexion (−9.6°), internal rotation (−3.0°), and lateral translation (−0.3 mm), while the patients with isolated CAI predominantly demonstrated reductions in internal rotation (−3.1°) and lateral translation (−0.4 mm). These findings can not only provide potential biomechanical targets for distinguishing between patients with CAI with and without an OLT, but also allow for tailored treatment regimens. This reduced ankle ROM may also be attributed to the presence of pain-related kinesiophobia in the ankle instability population, as a structural neuroimaging study indicated that individuals with CAI have significantly reduced mean gray matter density in the prefrontal cortex and periaqueductal gray matter. However, the precise neurological mechanisms remain to be clarified (38, 39). Ankle kinematics in patients with OLTs were similar to those of patients with ankle osteoarthritis (40). In the future, rehabilitative regimens for patients with OLTs that target ankle ROM and incorporate pain scores to measure kinesiophobia or psychological barriers may improve patient outcomes and avoid progression to ankle osteoarthritis.

Level walking is the most common physical activity in daily life. The kinematic changes during gait after an ATFL injury, with or without an OLT, can have an adverse effect on the ankle joint and can result in osteoarthritis (41). The ATFL is a stabilizer of the ankle joint. If it fails, the talus can rotate and translate unrestrictedly, leading to an alteration of the contact point between the talus and the calcaneus, increasing stress (35, 42). High-stress contact pain from ankle inversion and internal rotation is reduced by a coping strategy of delayed activation of the lateral fibular muscles in the early stance gait phase (17). Additionally, finite element analysis indicated that patients with OLTs have increased articular surface contact stress (43). Hence, some authors have extrapolated that these increased staccato contact stresses may be a potential biomechanical mechanism leading to focal ankle osteochondral lesions in patients with CAI (44). To reduce the risk of misdiagnosis and delayed treatment for patients with OLTs, a better understanding of ankle kinematics during walking is necessary.

There were several potential limitations in this study. First, we did not consider the distribution of different locations (medial vs. lateral) of OLT injuries, which limits the generalizability of the conclusions. Subgroups should be distinguished to further investigate the transition from CAI to OLT or even ankle osteoarthritis. Second, there was no grouping of the duration of the OLTs. Acute and chronic OLTs may result in different abnormal kinematic compensations. Third, the details of local osteoarticular motion were not captured; however, the Opti-Ankle system in this study can satisfy the needs of clinical testing (26). Fourth, the lack of a force plate to record data for inverse dynamics resulted in the relationship between ankle loading and lateral translation being unclear. Fifth, although the subjects underwent adequate familiarization (5 min) to ensure natural movement patterns, treadmill walking may impose kinematic constraints and alter one’s natural gait variability compared to overground conditions, which may reduce the validity of the findings. Finally, it should be acknowledged that this study did not account for the bias in results that may arise from having different evaluators; prospective cohort studies should be conducted by various professionals to enhance methodological rigor in the future.

## Conclusion

This study is the first to systematically characterize 6-DOF ankle kinematics in patients with MRI-confirmed CAI with and without an OLT during level walking. The results show that the addition of an OLT exacerbates kinematic abnormalities beyond isolated CAI, leading to significantly reduced plantarflexion ROM (9.6°) and limited distal translation in critical gait phases. These variables may serve as possible clinical screening tools for early OLT detection in CAI populations, potentially reducing diagnostic delays and progressive cartilage degeneration.

## Data Availability

The raw data supporting the conclusions of this article will be made available by the authors, without undue reservation.
